# Pioglitazone as a novel therapeutic approach in chronic granulomatous disease

**DOI:** 10.1016/j.jaci.2016.01.033

**Published:** 2016-06

**Authors:** Maddalena Migliavacca, Andrea Assanelli, Francesca Ferrua, Maria Pia Cicalese, Alessandra Biffi, Marta Frittoli, Paolo Silvani, Giovanna Chidini, Edoardo Calderini, Anna Mandelli, Anna Camporesi, Raffaella Milani, Giada Farinelli, Roberto Nicoletti, Fabio Ciceri, Alessandro Aiuti, Maria Ester Bernardo

**Affiliations:** a San Raffaele Telethon Institute for Gene Therapy (TIGET), Pediatric Immunohematology and Bone Marrow Transplantation Unit, San Raffaele Scientific Institute, Milan, Italy; b Hematology and Bone Marrow Transplantation Unit, San Raffaele Scientific Institute, Milan, Italy; c Department of Anesthesia and Critical Care, San Raffaele Hospital, Milan, Italy; d Pediatric Intensive Care Unit, Department of Anesthesia and Critical Care, Fondazione IRCCS Ca' Granda, Ospedale Maggiore Policlinico, Milan, Italy; e Pediatric Anesthesia and Intensive Care, Children Hospital “V.Buzzi”, Milan, Italy; f Cytometry Laboratory, San Raffaele Scientific Institute, Milan, Italy; g San Raffaele Telethon Institute for Gene Therapy (TIGET), San Raffaele Scientific Institute, Milan, Italy; h Department of Radiology, San Raffaele Scientific Institute, Milan, Italy

To the Editor:

Chronic granulomatous disease (CGD) is a rare genetic disease caused by defects in genes encoding the subunits of the nicotinamide adenine dinucleotide phosphate oxidase complex.^1^ Accordingly, patients with CGD are affected by life-threatening bacterial and fungal infections, as well as extensive tissue granuloma formation. Most frequently, CGD is caused by mutations in the X-chromosomal *CYBB* gene, which encodes for the gp91phox subunit.^2^ X-chromosome–linked CGD (X-CGD) generally produces a severe phenotype, with a mortality rate of 3% to 5% per year despite state-of-the-art prophylaxis and intensive multimodal treatment.^3^ Hematopoietic stem cell transplantation (HSCT) represents a curative treatment for patients with X-CGD with excellent survival thanks to recent improvements in HSCT protocols.^4^ The treatment of patients without an HLA-matched donor and active infections/inflammatory complications remains challenging and requires novel approaches.^5^

Recently, pioglitazone, a peroxisome proliferator–activated receptor gamma agonist approved for type 2 diabetes, was reported in this journal to induce mitochondrial (mt) reactive oxygen species (ROS) production in mice with X-CGD. Restored phagocytes demonstrated significantly enhanced killing of *Staphylococcus aureus in vitro* and *in vivo*.^6^

We report, for the first time, the use of pioglitazone as a novel therapeutic approach in a 5-month-old boy with X-CGD experiencing multiple severe infections.

At 1 month of life, the patient developed a cutaneous abscess, fever, and respiratory distress requiring artificial ventilation in pediatric intensive care unit. During the hospitalization, he developed 3 septic shocks, secondary to a peritonsillar abscess, *Candida albicans*, and *Staphylococcus epidermidis* dissemination, respectively. Thoracic computed tomography scan showed multiple pulmonary abscesses (Fig 1, *A*). Because of the susceptibility to severe infections with organ damage and failure to thrive, he was investigated for a potential congenital immunodeficiency. Dihydrorhodamine (DHR) fluorescence showed impaired response in his peripheral blood and a mutation of the *CYBB* gene was identified (c.483+1G>T). This mutation, in literature, is associated with a X-linked CGD form with undetectable level of gp91phox protein.^7^

Because of the underlying general conditions of the patient with persistent pulmonary distress requiring protracted noninvasive ventilation, severe delay in neuromuscular development, and failure to thrive, HSCT was postponed despite availability of an HLA-identical sibling.

Encouraged by the innovative findings on the effect of pioglitazone on ROS production and consequent protection from infection *in vitro* in human cells and *in vivo* in an animal model,^6^ we sought to investigate, for the first time to our knowledge in children, the potential beneficial effect of oral pioglitazone. After parent informed consent for off-label use, pioglitazone was administered at a starting dose of 1 mg/kg and given the absence of adverse effects was progressively increased up to 3 mg/kg (15 mg/daily). After 10 days of pioglitazone at target dose (3 mg/kg), the DHR test showed a percentage of granulocytes with increased DHR fluorescence (bright fluorescent cells = 12.90%; value before treatment 0.06%; Fig 2, *A* and *B*), albeit at a lower mean fluorescence intensity than normal donor cells. This effect was maintained in the following weeks until withdrawal (day+30), although at a lower level (day+17, 7.55%; day+25, 4.86%). Moreover, an overall shift in DHR of the whole population of patient's granulocytes was noted after pioglitazone treatment (for details, see Table E1 in this article's Online Repository at www.jacionline.org).

These findings paralleled well with clinical, radiological, and blood parameters. The patient experienced a progressive improvement in general clinical condition, respiratory parameters, and function with stable oxygen saturation under neurally adjusted ventilatory assist-noninvasive ventilation. Lung computed tomography scan performed on the day of pioglitazone withdrawal showed clear improvement (Fig 1, *B*). White blood cell (WBC) counts, as well as neutrophil counts, progressively decreased to normal levels (WBC, 13.9 × 109/L, and neutrophil, 8.2 × 109/L before pioglitazone; WBC, 6.8 × 109/L, and neutrophil, 2 × 109/L at pioglitazone withdrawal, respectively). C-Reactive protein level decreased from 24.4 mg/L to 13.1 mg/L before and after pioglitazone, respectively.

The clinical and radiologic amelioration allowed for HSCT; pioglitazone was withdrawn and after 1 week, conditioning regimen based on treosulfan/fludarabine/Thiotepa was administered to the patient.^8^ Graft versus host disease prophylaxis was conducted through administration of cyclosporin-A and short-term methotrexate. Neutrophil and platelet engraftment took place at day+17 and +32, respectively. The patient did not develop signs of graft versus host disease. He is currently at day+150 after HSCT in good clinical condition with donor chimerism above 90% on CD14^+^, CD15^+^, and CD3^+^ cells in peripheral blood. He does not require any respiratory support, he has a satisfactory oral food intake with weight gain, and he shows neuromuscular development improvement.

Although observed in a single child, pioglitazone might have played a role in the protection of the patient from further infections and in the improvement of his pulmonary situation, by reducing the inflammatory status and by partial restoration of host defense through induction of mtROS production in his granulocytes. This is also supported by the fact that concomitant medications were not increased, but rather reduced in intensity during pioglitazone treatment (for details, see Table E2 in this article's Online Repository at www.jacionline.org). Moreover, corticosteroids were never administered to the patient, neither before nor during pioglitazone treatment. Moreover, the patient did not develop any adverse effects related to pioglitazone administration, confirming the safety of this therapeutic approach. Considering data in female carriers, patients with HSCT, and improvement after gene therapy,^7^, ^9^ we speculate that even an enhancement in DHR fluorescence in a relatively small population of leukocytes might be the expression of a protective ROS production. Accordingly, Fernandez-Boyanapalli et al^6^ suggest that the mechanism responsible for peroxisome proliferator–activated receptor gamma agonist effect in mice with CGD is related to increased ROS production by mitochondria in a subpopulation of neutrophils, monocytes, and inflammatory macrophages. It remains to be determined which population is targeted by pioglitazone *in vivo*.

Presently our observations cannot provide information regarding long-term effects on mtROS production, but suggest that pioglitazone might be used as bridge treatment for these fragile chronic patients. Fernandez-Boyanapalli et al's findings and our experience on the use of pioglitazone provide relevant insights into the treatment of this rare disease especially for those patients without a prompt suitable matched donor or for whom the critical disease conditions force to postpone HSCT.

Further studies conducted through well-designed clinical trials are warranted to investigate the drug's precise mechanism of action, as well as to define the role of pioglitazone in the therapeutic armamentarium of patients with X-CGD.

## Figures and Tables

**Table E1:** Percentage of DHR bright and DHR positive granulocytes according to pioglitazone dose∗

Time point	Pioglitazone dose	DHR bright fluorescent cells (%)	DHR fluorescent cells (%)	MFI on total granulocytes (fold increase)
Before treatment	/	0.06	0.07	0.90
5 d of pioglitazone	1 mg/kg	0.20	0.25	0.92
10 d of pioglitazone	2 mg/kg	0.54	0.65	0.97
25 d of pioglitazone (d+10)	3 mg/kg	12.90	76.40	2.87
32 d of pioglitazone (d+17)	3 mg/kg	7.55	21.00	1.39
40 d of pioglitazone (d+25)	3 mg/kg	4.86	6.48	1.29
After HSCT	/	94.10	98.30	20.54

*MFI*, Mean fluorescent intensity.

∗ The fold increase in MFI is also depicted and is calculated as the ratio between the MFI on total granulocytes after stimulation with PMA and the MFI on unstimulated total granulocytes.

**Table E2:** Concomitant medications received by the patient during pioglitazone treatment∗

Concomitant medications	Dose per day	State at pioglitazone start	State at pioglitazone stop
Linezolid IV	30 mg/kg	Ongoing	Stopped 7 d after pioglitazone start
Piperacillin-tazobactam IV	300 mg/kg	Ongoing	Stopped 10 d after pioglitazone start
Amoxicillin-clavulanic acid PO	50 mg/kg	Started 10 d after pioglitazone initiation	Ongoing
Trimethoprim-sulphamethoxazole PO	5-25 mg/kg	Ongoing	Ongoing
Voriconazole IV	8 mg/kg	Ongoing	Stopped 6 d after pioglitazone start
Itraconazole PO	10 mg/kg	Started 6 d after pioglitazone initiation	Ongoing
Noninvasive ventilation	NA	Ongoing	Ongoing

*IV*, Intravenous; *NA*, not applicable; *PO*, *per os* (by mouth).

∗ Days are expressed in relation to the first day of pioglitazone administration.

**Fig 1:**
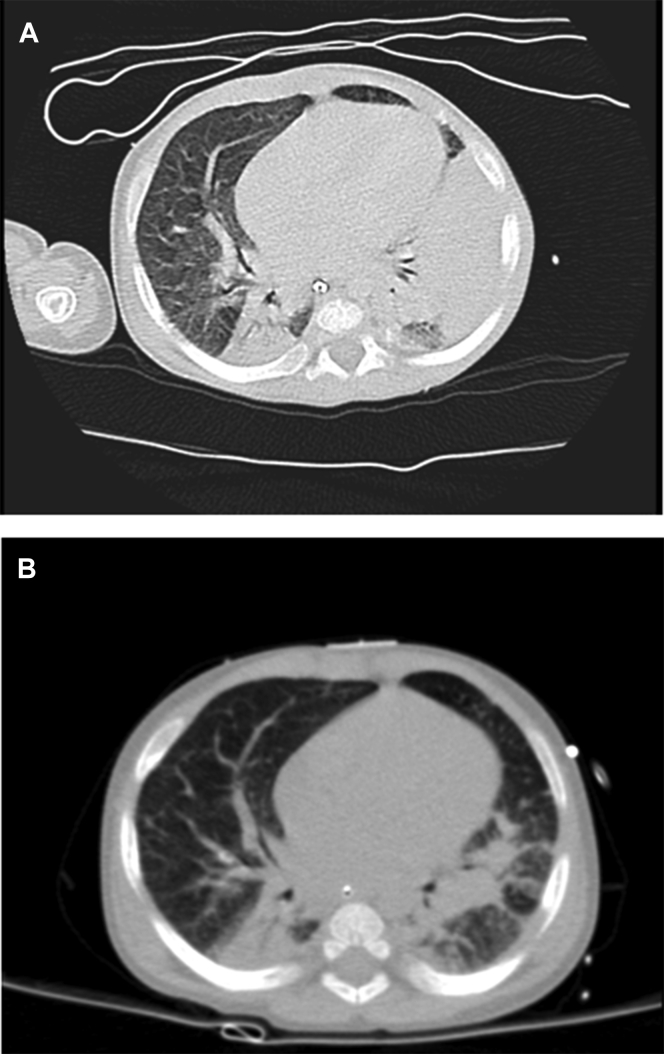
Chest computed tomography scan before **(A)** and after **(B)** pioglitazone treatment. Fig 1, *A*, Large left pulmonary consolidation involving the inferior lobe and, partly, the dorsal and lingular segments of the superior lobe, with multiple internal air bronchograms: pneumonia. Similar pulmonary consolidation is present in the right lung and involves the dorsal segments of the inferior lobe. Fig 1, *B*, Remarkable reduction in the areas of consolidation in the left and right lungs; residual pulmonary lesions are visible in the left and right inferior lobes.

**Fig 2:**
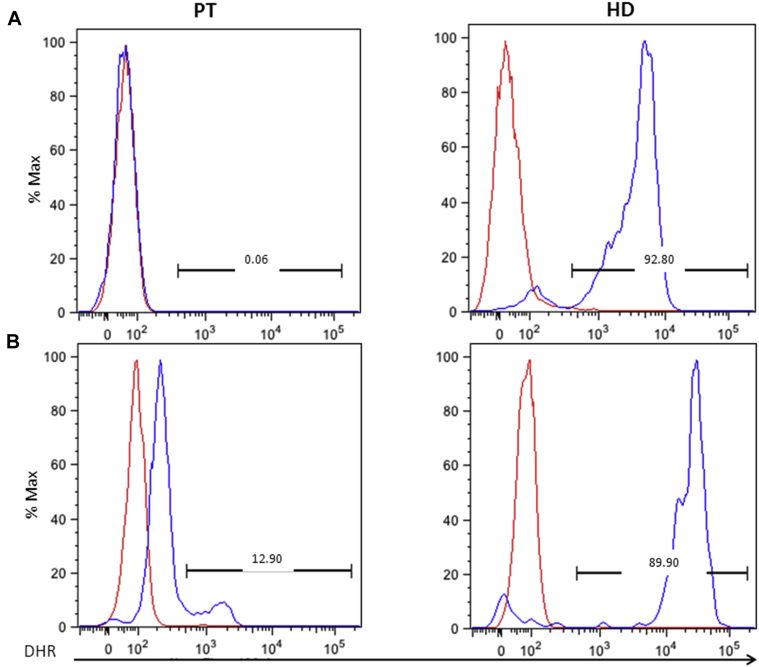
DHR fluorescence after stimulation with the protein kinase C activator, phorbol 12-myristate 13-acetate (PMA), before and after pioglitazone treatment for the patient (PT) and a healthy donor (HD). DHR analysis was performed on granulocytes by Phagoburts (BDBiosciences, Milan, Italy) according to manufacturer's instructions and analyzed by flow-cytometry. *Red line* represents the unstimulated condition; *blue line* indicates the PMA-stimulated condition. **A,** DHR results before pioglitazone treatment; representative histograms for neutrophils show 0.06% response. MFI on total granulocytes (fold increase): 0.90 in patient vs 43.02 in HD. **B,** DHR results at day+10 at target dose demonstrate 12.90% granulocytes with increased DHR fluorescence. MFI on total granulocytes (fold increase): 2.87 in patient vs 149.7 in HD. *MFI*, Mean fluorescent intensity.
